# Supplementary figures and images for: RNA sequencing revealed the multi-stage transcriptome transformations during the development of gallbladder cancer associated with chronic inflammation
Source: PLoS One. 2023 Mar 30;18(3):e0283770. doi: 10.1371/journal.pone.0283770 (PMC10062614; doi:10.1371/journal.pone.0283770)

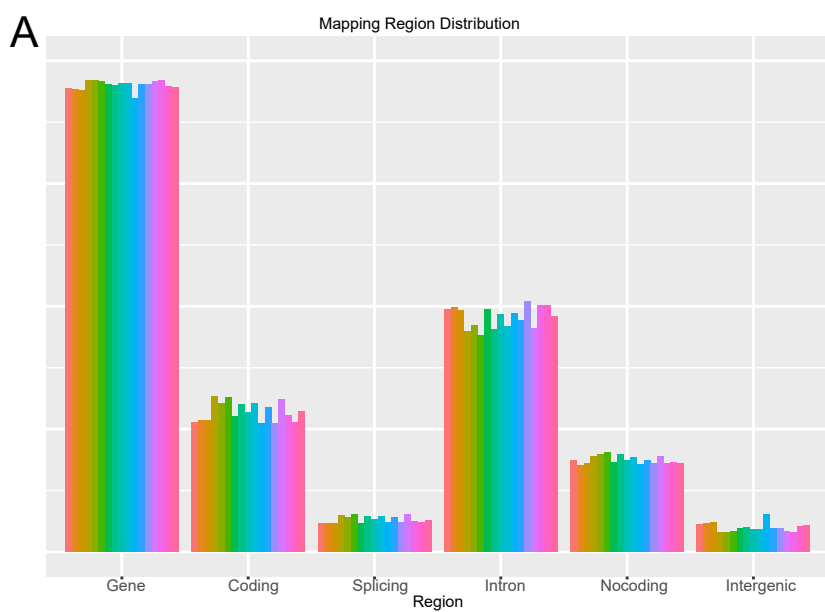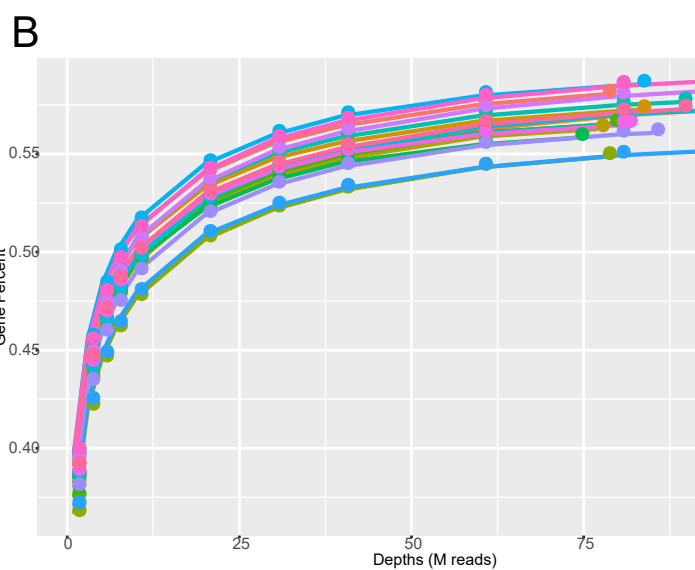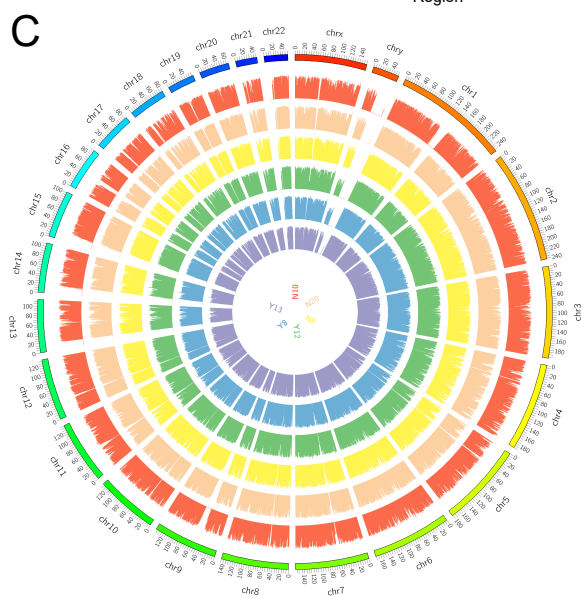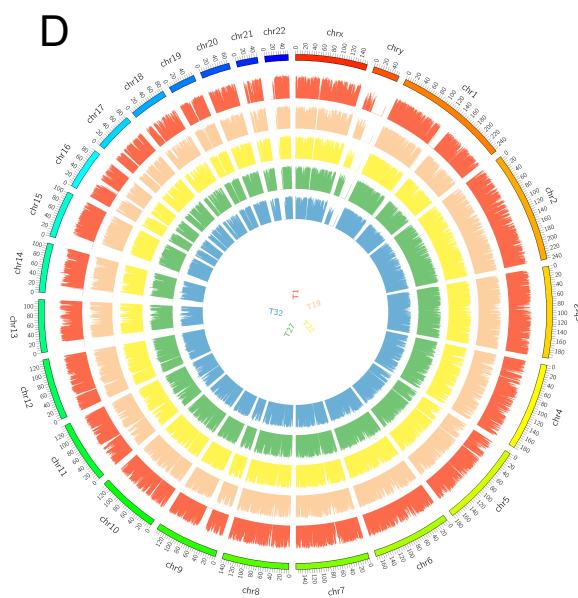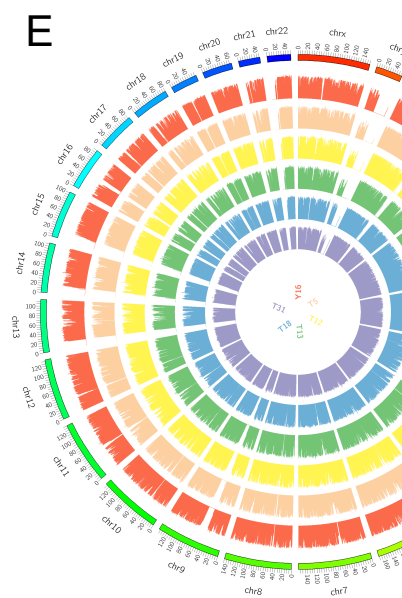

Supplement: S1 Fig — (A) Compared with the human genome, the ratio of reads aligned to gene regions, coding regions, splice sites, introns and non-coding regions was normal. (B) Saturation analysis indicated that the amount of sequencing was sufficient. (C) The sequencing results of samples N10, N20, N8, Y12, Y8, Y13 well covered the genome. (D) The sequencing results of samples T1 T19 T22 T27 T32 well covered the genome. (E) The sequencing results of samples Y16 T5 T12 T13 T18 T31 well covered the genome. (PDF) [file pone.0283770.s001.pdf]

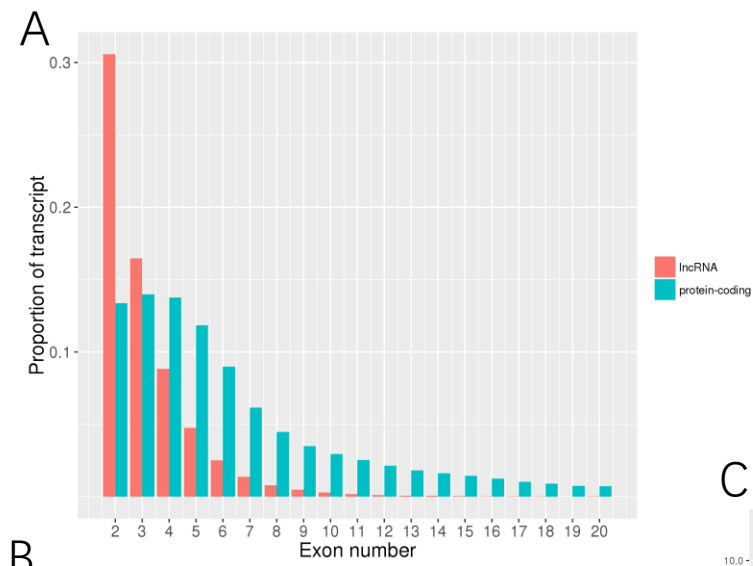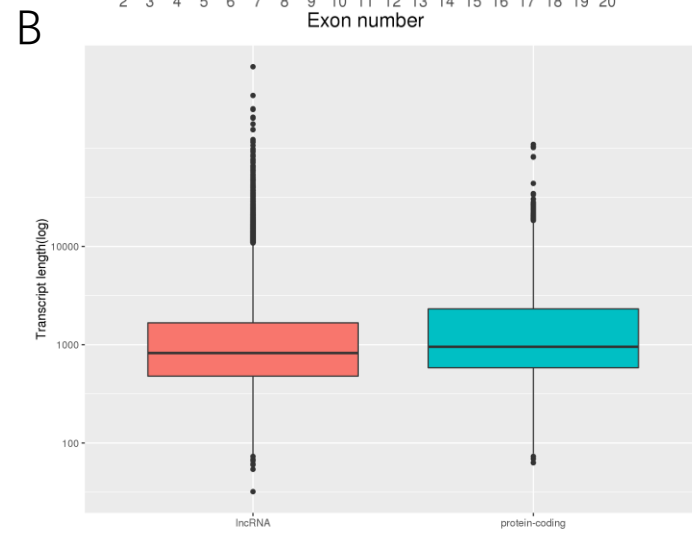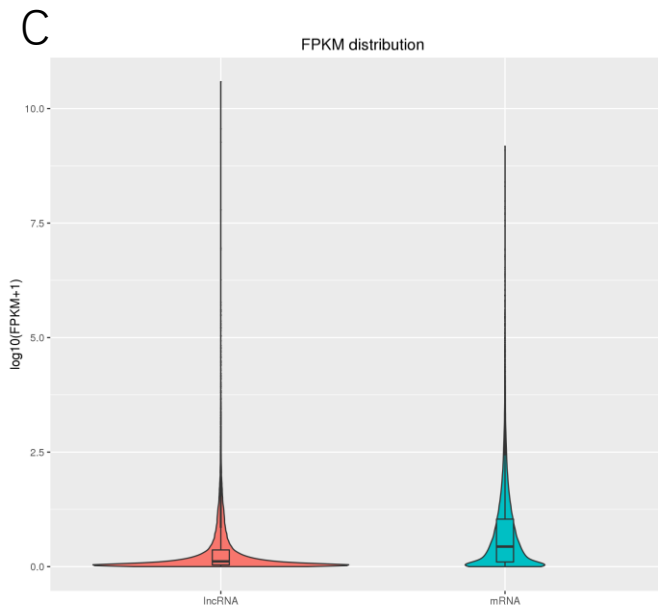

Supplement: S2 Fig — (A) Comparison of the number of exons between lncRNAs and mRNAs. (B) Comparison of the length distribution of lncRNAs and mRNAs. (C) Comparison of the expression levels of lncRNAs and mRNAs: take the average of the expression values of each transcript of lncRNA and mRNA, and draw the box plot with the log10 (FPKM+1) values. (PDF) [file pone.0283770.s002.pdf]

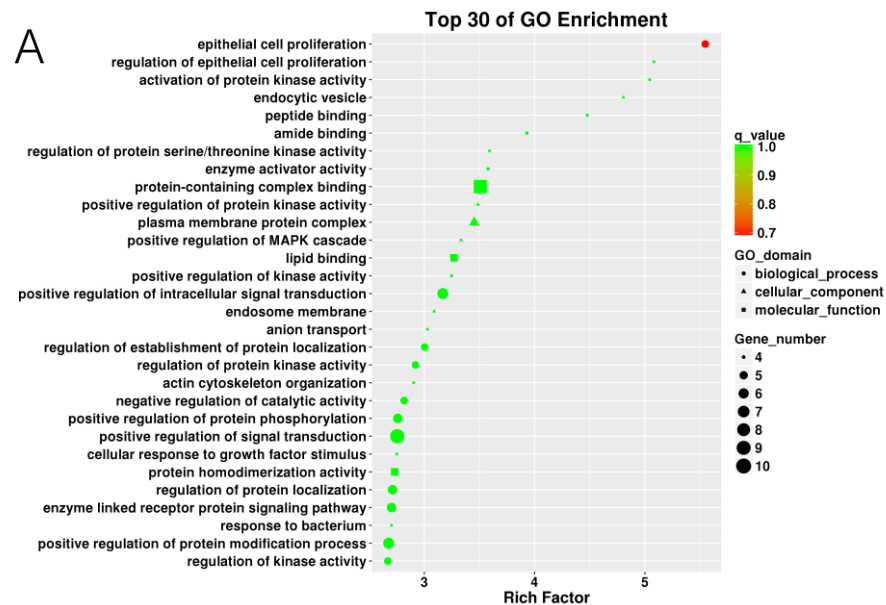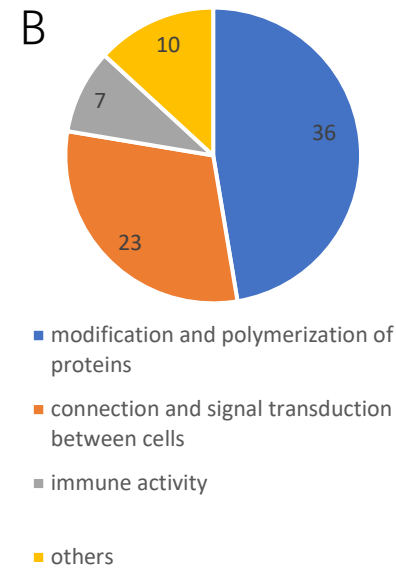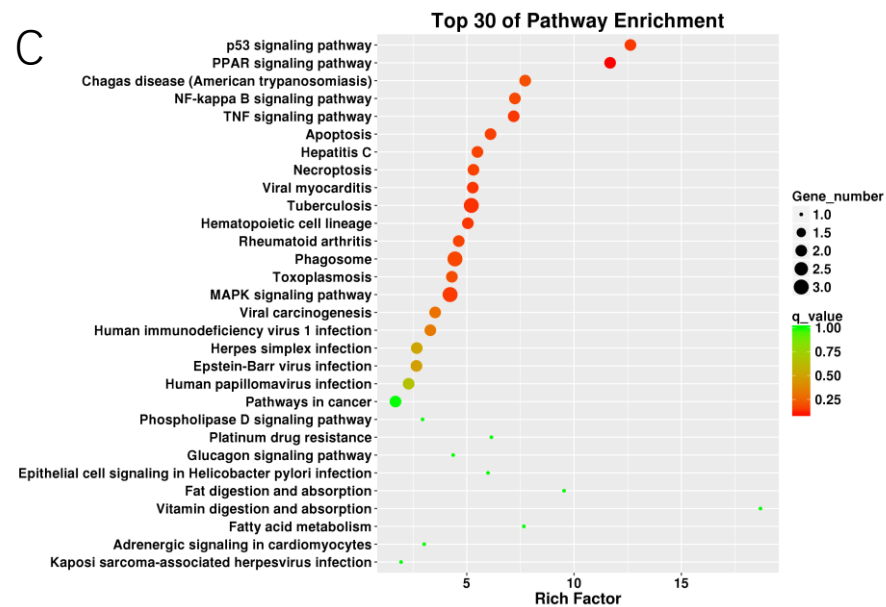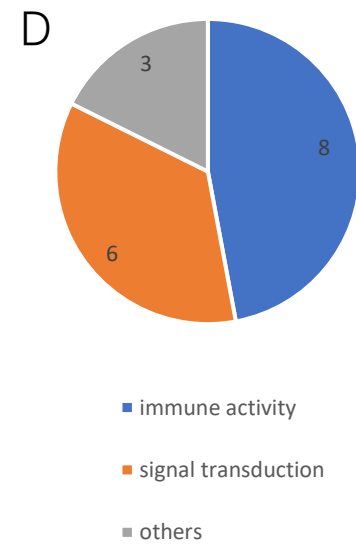

Supplement: S4 Fig — (A) The top 30 GO terms with a high degree of enrichment, the shapes of icons represent different GO categories, the size represents the number of target genes of differentially expressed lncRNAs, the color depth represents the size of the q-value, and the X axis indicates the value of rich factor. (B) The 76 GO terms with p-value ≤ 0.05 were further classified, numbers on the graph represent the number of GO terms corresponding to the category. (C)The top 30 KEGG terms with a high degree of enrichment. (D) The 17 KEGG terms with p-value ≤ 0.05 were further classified, numbers on the graph represent the number of KEGG terms corresponding to the category. (PDF) [file pone.0283770.s004.pdf]

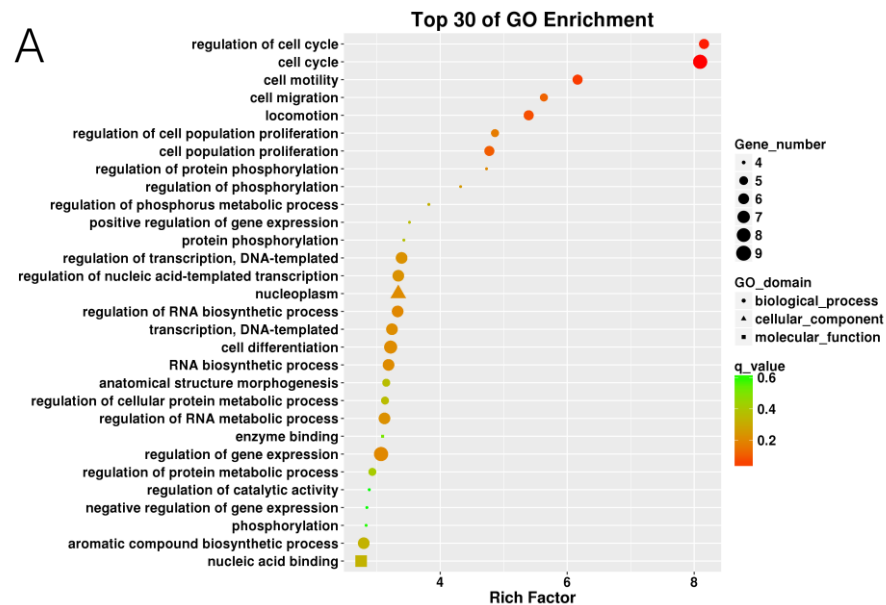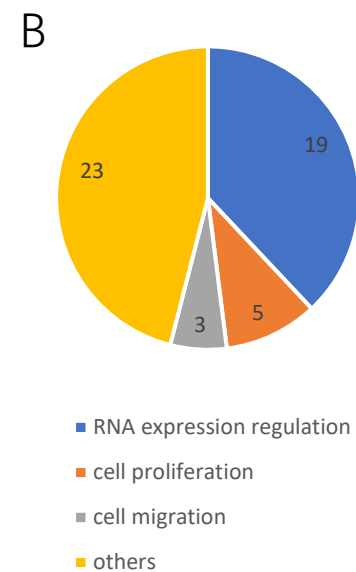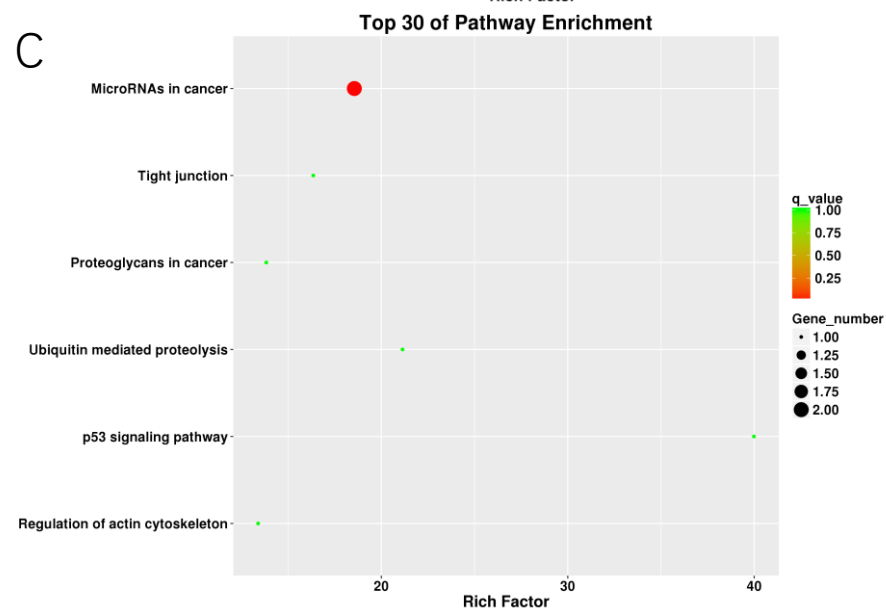

Supplement: S5 Fig — (A) The top 30 GO terms with a high degree of enrichment, the shapes of icons represent different GO categories, the size represents the number of target genes of differentially expressed lncRNAs, the color depth represents the size of the q-value, and the X axis indicates the value of rich factor. (B) The 50 GO terms with p-value ≤ 0.05 were further classified, numbers on the graph represent the number of GO terms corresponding to the category. (C) The top 30 KEGG terms with a high degree of enrichment. (PDF) [file pone.0283770.s005.pdf]
