# Supplementary material for: RNA sequencing revealed the multi-stage transcriptome transformations during the development of gallbladder cancer associated with chronic inflammation
Source: PLoS One. 2023 Mar 30;18(3):e0283770. doi: 10.1371/journal.pone.0283770 (PMC10062614; doi:10.1371/journal.pone.0283770)
Supplement: S3 Fig — (A) The top 30 GO terms with a high degree of enrichment, the shapes of icons represent different GO categories, the size represents the number of differentially expressed target genes of differentially expressed lncRNAs contained by this GO term, the color depth represents the size of the q-value, the X axis indicates the value of rich factor. (B) The 28 GO terms with q-value ≤ 0.05 were further classified, numbers on the graph represent the number of GO terms corresponding to the category. (C) The top 30 KEGG terms with a high degree of enrichment. (D) The 16 KEGG terms with p-value ≤ 0.05 were further classified, numbers on the graph represent the number of KEGG items corresponding to the category. (PDF) [file pone.0283770.s003.pdf]

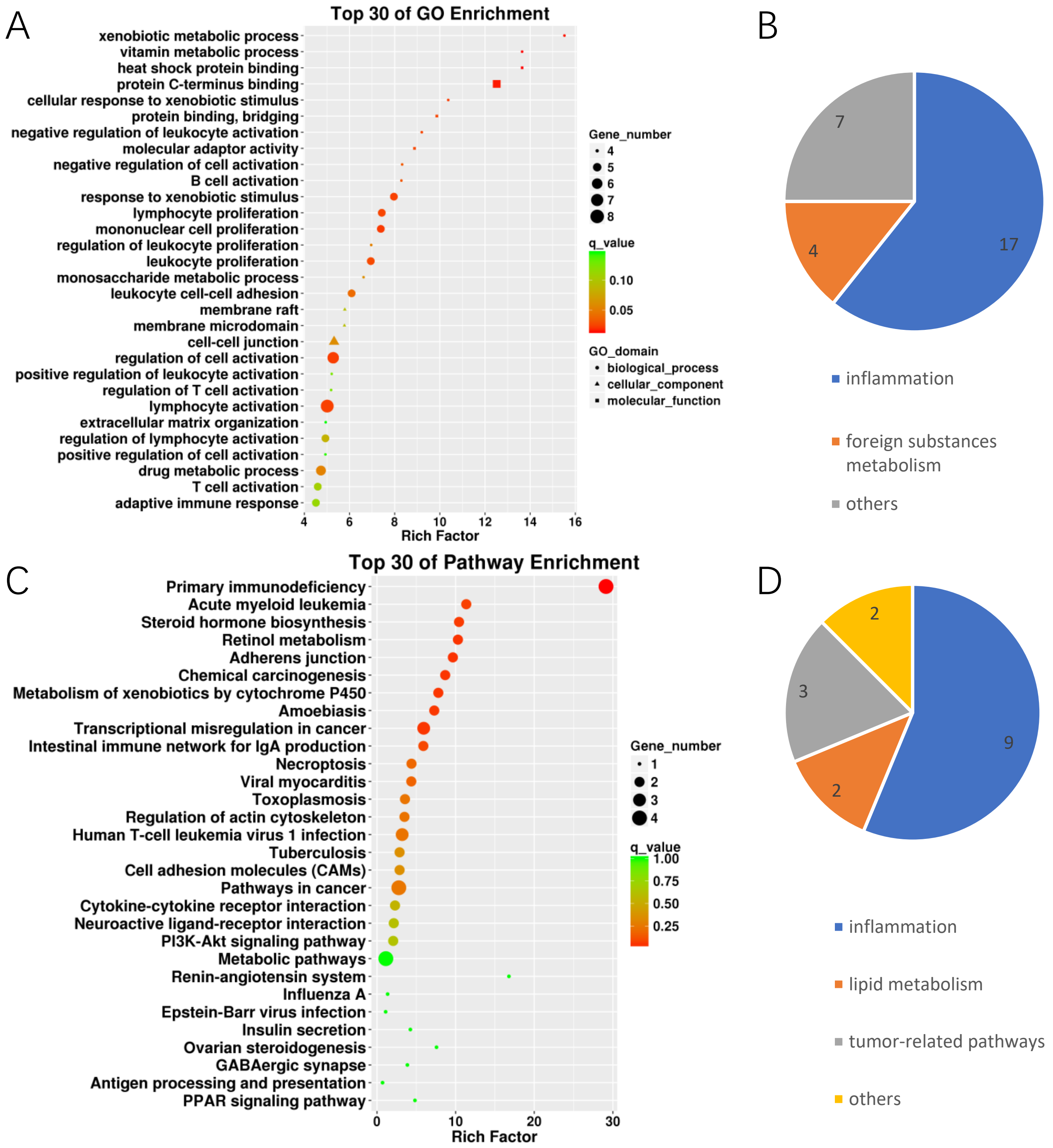
A
Top 30 of GO Enrichment
xenobiotic metabolic process
vitamin metabolic process
heat shock protein binding
protein C-terminus binding
cellular response to xenobiotic stimulus
protein binding, bridging
negative regulation of leukocyte activation
molecular adaptor activity
negative regulation of cell activation
B cell activation
response to xenobiotic stimulus
lymphocyte proliferation
mononuclear cell proliferation
regulation of leukocyte proliferation
leukocyte proliferation
monosaccharide metabolic process
leukocyte cell-cell adhesion
membrane raft
membrane microdomain
cell-cell junction
regulation of cell activation
positive regulation of leukocyte activation
regulation of T cell activation
lymphocyte activation
extracellular matrix organization
regulation of lymphocyte activation
positive regulation of cell activation
drug metabolic process
T cell activation
adaptive immune response
4 6 8 10 12 14 16
Rich Factor
Gene_number
4
5
6
7
8
q_value
0.10
0.05
GO_domain
biological_process
cellular_component
molecular_function
B
7
4
17
inflammation
foreign substances metabolism
others
C
Top 30 of Pathway Enrichment
Primary immunodeficiency
Acute myeloid leukemia
Steroid hormone biosynthesis
Retinol metabolism
Adherens junction
Chemical carcinogenesis
Metabolism of xenobiotics by cytochrome P450
Amoebiasis
Transcriptional misregulation in cancer
Intestinal immune network for IgA production
Necroptosis
Viral myocarditis
Toxoplasmosis
Regulation of actin cytoskeleton
Human T-cell leukemia virus 1 infection
Tuberculosis
Cell adhesion molecules (CAMs)
Pathways in cancer
Cytokine-cytokine receptor interaction
Neuroactive ligand-receptor interaction
PI3K-Akt signaling pathway
Metabolic pathways
Renin-angiotensin system
Influenza A
Epstein-Barr virus infection
Insulin secretion
Ovarian steroidogenesis
GABAergic synapse
Antigen processing and presentation
PPAR signaling pathway
0 10 20 30
Rich Factor
Gene_number
1
2
3
4
q_value
1.00
0.75
0.50
0.25
D
2
3
9
2
inflammation
lipid metabolism
tumor-related pathways
others
