# Supplementary material for: RNA sequencing revealed the multi-stage transcriptome transformations during the development of gallbladder cancer associated with chronic inflammation
Source: PLoS One. 2023 Mar 30;18(3):e0283770. doi: 10.1371/journal.pone.0283770 (PMC10062614; doi:10.1371/journal.pone.0283770)
Supplement: S1 Table — (DOCX) [file pone.0283770.s006.docx]

**S1 Table. Clinicopathological data of normal gallbladder and gallbladder with chronic inflammation**

| Number | Sex | Age | Gallstone | Disease | Operation | Pathology |
| --- | --- | --- | --- | --- | --- | --- |
| N8 | male | 53 | no | pancreas tumor | pancreaticoduodenectomy | normal |
| N10 | male | 69 | no | pancreas tumor | pancreaticoduodenectomy | normal |
| N20 | male | 49 | no | hepatic hemangioma | right hepatectomy | normal |
| Y8 | female | 36 | yes | cholecystitis | laparoscopic cholecystectomy | chronic inflammation |
| Y12 | female | 71 | yes | cholecystitis | laparoscopic cholecystectomy | chronic inflammation |
| Y13 | male | 76 | yes | cholecystitis | laparoscopic cholecystectomy | chronic inflammation |
| Y16 | male | 58 | yes | cholecystitis | laparoscopic cholecystectomy | chronic inflammation |
