# Supplementary material for: RNA sequencing revealed the multi-stage transcriptome transformations during the development of gallbladder cancer associated with chronic inflammation
Source: PLoS One. 2023 Mar 30;18(3):e0283770. doi: 10.1371/journal.pone.0283770 (PMC10062614; doi:10.1371/journal.pone.0283770)
Supplement: S2 Table — (DOCX) [file pone.0283770.s007.docx]

**S2 Table. Clinicopathological data of gallbladder cancer**

| Number | Sex | Age | T | N | M | Stage | Pathology |
| --- | --- | --- | --- | --- | --- | --- | --- |
| T5 | female | 70 | 2 | 0 | 0 | Ⅱ | adenocarcinoma |
| T12 | female | 54 | 1 | 0 | 0 | Ⅰ | adenocarcinoma |
| T13 | female | 69 | 2 | 0 | 0 | Ⅱ | adenocarcinoma |
| T18 | male | 71 | 1 | 0 | 0 | Ⅰ | adenocarcinoma |
| T31 | female | 66 | 2 | 0 | 0 | Ⅱ | adenocarcinoma |
| T1 | female | 65 | 4 | 2 | 0 | ⅣB | adenocarcinoma |
| T19 | male | 71 | 4 | 2 | 0 | ⅣB | adenocarcinoma |
| T22 | female | 67 | 4 | 2 | 0 | ⅣB | adenocarcinoma |
| T27 | male | 62 | 4 | 2 | 0 | ⅣB | adenocarcinoma |
| T32 | female | 67 | 3 | 2 | 0 | ⅣB | adenocarcinoma |
