# Supplementary material for: RNA sequencing revealed the multi-stage transcriptome transformations during the development of gallbladder cancer associated with chronic inflammation
Source: PLoS One. 2023 Mar 30;18(3):e0283770. doi: 10.1371/journal.pone.0283770 (PMC10062614; doi:10.1371/journal.pone.0283770)
Supplement: S3 Table — (DOCX) [file pone.0283770.s008.docx]

**S3 Table. Reagents for library construction and quality inspection**

| **Martials** | **Manufacturer** | **Number** |
| --- | --- | --- |
| VAHTS Universal V6 RNA-seq Library Prep Kit for Illumina® | Vazyme | NR604-02 |
| VAHTS Ribo-off rRNA Depletion Kit (Human/Mouse/Rat) | Vazyme | N406-02 |
| VAHTS DNA Clean Beads | Vazyme | N411-03 |
| Qubit™ dsDNA HS Assay Kit | Invitrogen | Q32854 |
| Agilent High Sensitivity DNA Kit | Agilent | 5067-4626 |
