# Supplementary material for: RNA sequencing revealed the multi-stage transcriptome transformations during the development of gallbladder cancer associated with chronic inflammation
Source: PLoS One. 2023 Mar 30;18(3):e0283770. doi: 10.1371/journal.pone.0283770 (PMC10062614; doi:10.1371/journal.pone.0283770)
Supplement: S4 Table — (DOCX) [file pone.0283770.s009.docx]

**S4 Table. Quality inspection results of library**

| **No.** | **Sample Name** | **Index No.** | **Con.**  **(ng/μL)** | **Peak Lenth.**  **(bp)** |
| --- | --- | --- | --- | --- |
| 1 | N8 | V87 | 25.2 | 390 |
| 2 | Y13 | V88 | 24.8 | 390 |
| 3 | T5 | V89 | 24.6 | 390 |
| 4 | T12 | V90 | 26.0 | 390 |
| 5 | T13 | V91 | 25.8 | 390 |
| 6 | T18 | V92 | 24.4 | 390 |
| 7 | T31 | V93 | 26.8 | 390 |
| 8 | T1 | V94 | 26.4 | 390 |
| 9 | T19 | V95 | 27.8 | 390 |
| 10 | T22 | V96 | 24.2 | 390 |
| 11 | T27 | V1 | 24.0 | 390 |
| 12 | N10 | V2 | 26.0 | 390 |
| 13 | N20 | V3 | 22.2 | 390 |
| 14 | Y8 | V4 | 25.0 | 390 |
| 15 | Y12 | V5 | 25.0 | 390 |
| 16 | Y16 | V6 | 24.8 | 390 |
| 17 | T11 | V7 | 27.0 | 390 |
| 18 | T24 | V8 | 23.8 | 390 |
| 19 | T30 | V9 | 23.6 | 390 |
| 20 | T32 | V10 | 23.4 | 390 |
