# Supplementary material for: RNA sequencing revealed the multi-stage transcriptome transformations during the development of gallbladder cancer associated with chronic inflammation
Source: PLoS One. 2023 Mar 30;18(3):e0283770. doi: 10.1371/journal.pone.0283770 (PMC10062614; doi:10.1371/journal.pone.0283770)
Supplement: S5 Table — (DOCX) [file pone.0283770.s010.docx]

**S5 Table. Inspection results of sequencing data**

| **Sample Name** | **Seq. type** | **Orientation** | **Raw reads (M)** | **Raw bases(G)** | **Q20 ratio (%)** |
| --- | --- | --- | --- | --- | --- |
| N10 | mRNA | Forward/Reverse | 93.91 | 14.09 | 97.58% |
| N20 | mRNA | Forward/Reverse | 123.88 | 18.58 | 97.43% |
| N8 | mRNA | Forward/Reverse | 92.91 | 13.94 | 97.98% |
| T1 | mRNA | Forward/Reverse | 86.30 | 12.94 | 98.09% |
| T12 | mRNA | Forward/Reverse | 89.12 | 13.37 | 97.36% |
| T13 | mRNA | Forward/Reverse | 88.51 | 13.28 | 97.94% |
| T18 | mRNA | Forward/Reverse | 84.87 | 12.73 | 97.86% |
| T19 | mRNA | Forward/Reverse | 91.61 | 13.74 | 98.11% |
| T22 | mRNA | Forward/Reverse | 101.65 | 15.25 | 98.08% |
| T27 | mRNA | Forward/Reverse | 104.87 | 15.73 | 97.73% |
| T31 | mRNA | Forward/Reverse | 90.78 | 13.62 | 98.00% |
| T32 | mRNA | Forward/Reverse | 104.76 | 15.71 | 98.08% |
| T5 | mRNA | Forward/Reverse | 96.29 | 14.44 | 97.94% |
| Y12 | mRNA | Forward/Reverse | 112.70 | 16.90 | 97.51% |
| Y13 | mRNA | Forward/Reverse | 93.22 | 13.98 | 97.88% |
| Y16 | mRNA | Forward/Reverse | 110.28 | 16.54 | 97.54% |
| Y8 | mRNA | Forward/Reverse | 99.42 | 14.91 | 97.64% |
