# Supplementary material for: RNA sequencing revealed the multi-stage transcriptome transformations during the development of gallbladder cancer associated with chronic inflammation
Source: PLoS One. 2023 Mar 30;18(3):e0283770. doi: 10.1371/journal.pone.0283770 (PMC10062614; doi:10.1371/journal.pone.0283770)
Supplement: S6 Table — (DOCX) [file pone.0283770.s011.docx]

**S6 Table. Preprocessed statistics of sequencing**

| **Sample ID** | **Raw reads** | **Clean reads** | **Clean ratio** | **rRNA trimed** | **rRNA ratio** | **No rRNA pair** |
| --- | --- | --- | --- | --- | --- | --- |
| N10 | 93913288 | 80817580 | 86.06% | 80180308 | 0.79% | 78432602 |
| N20 | 123881794 | 108813367 | 87.84% | 108099904 | 0.66% | 106128366 |
| N8 | 92910162 | 85180618 | 91.68% | 84614978 | 0.66% | 83207008 |
| T1 | 86296474 | 79621040 | 92.26% | 78674365 | 1.19% | 77641636 |
| T12 | 89126302 | 80732538 | 90.58% | 80188939 | 0.67% | 78399530 |
| T13 | 88513202 | 81590288 | 92.18% | 80994217 | 0.73% | 79649690 |
| T18 | 84868934 | 76800210 | 90.49% | 75984691 | 1.06% | 74593372 |
| T19 | 91611974 | 83699928 | 91.36% | 82469896 | 1.47% | 81142248 |
| T22 | 101654112 | 92743324 | 91.23% | 91467382 | 1.38% | 89866080 |
| T27 | 104868064 | 96087049 | 91.63% | 95145410 | 0.98% | 93451314 |
| T31 | 90784898 | 85305569 | 93.96% | 84391497 | 1.07% | 83241384 |
| T32 | 104762620 | 96682841 | 92.29% | 95764848 | 0.95% | 94461300 |
| T5 | 96288848 | 88508104 | 91.92% | 87478517 | 1.16% | 85965440 |
| Y12 | 112699178 | 102187688 | 90.67% | 100785738 | 1.37% | 98736526 |
| Y13 | 93217200 | 85018133 | 91.20% | 82895524 | 2.50% | 81529274 |
| Y16 | 110276666 | 99928631 | 90.62% | 98847290 | 1.08% | 97105652 |
| Y8 | 99421262 | 92087921 | 92.62% | 91270581 | 0.89% | 89870108 |

Clean ratio = (Clean reads/Raw reads)%; rRNA ratio=[(Clean reads - rRNA trimed)/ Clean reads]%
