# Supplementary material for: RNA sequencing revealed the multi-stage transcriptome transformations during the development of gallbladder cancer associated with chronic inflammation
Source: PLoS One. 2023 Mar 30;18(3):e0283770. doi: 10.1371/journal.pone.0283770 (PMC10062614; doi:10.1371/journal.pone.0283770)
Supplement: S7 Table — (DOCX) [file pone.0283770.s012.docx]

**S7 Table. Genome mapping results**

| **Samples_ID** | **All reads** | **Mapped reads** | **Mapped**  **Pair**  **Reads** | **Mapped**  **broken-pair**  **reads** | **Mapped**  **Unique**  **reads** | **Mapped**  **Multi**  **reads** | **Mapping ratio** |
| --- | --- | --- | --- | --- | --- | --- | --- |
| N10 | 78432602 | 75536809 | 74078914 | 1457895 | 75137832 | 398977 | 96.31% |
| N20 | 106128366 | 102578289 | 100281280 | 2297009 | 101936929 | 641360 | 96.65% |
| N8 | 83207008 | 80759385 | 79413810 | 1345575 | 80416300 | 343085 | 97.06% |
| T12 | 78399530 | 76017517 | 74720872 | 1296645 | 75623176 | 394341 | 96.96% |
| T13 | 79649690 | 77552132 | 76294358 | 1257774 | 77226224 | 325908 | 97.37% |
| T18 | 74593372 | 71942559 | 70536450 | 1406109 | 71571244 | 371315 | 96.45% |
| T19 | 81142248 | 78490086 | 77135184 | 1354902 | 78094540 | 395546 | 96.73% |
| T1 | 77641636 | 75299878 | 73960106 | 1339772 | 74909277 | 390601 | 96.98% |
| T22 | 89866080 | 87209321 | 85825248 | 1384073 | 86829349 | 379972 | 97.04% |
| T27 | 93451314 | 90497604 | 88807888 | 1689716 | 90000744 | 496860 | 96.84% |
| T31 | 83241384 | 80533381 | 79147470 | 1385911 | 80192837 | 340544 | 96.75% |
| T32 | 94461300 | 91969996 | 90597242 | 1372754 | 91595834 | 374162 | 97.36% |
| T5 | 85965440 | 83372348 | 81920526 | 1451822 | 82994756 | 377592 | 96.98% |
| Y12 | 98736526 | 95292660 | 93412522 | 1880138 | 94759980 | 532680 | 96.51% |
| Y13 | 81529274 | 78606837 | 77307710 | 1299127 | 78233675 | 373162 | 96.42% |
| Y16 | 97105652 | 94016479 | 92214898 | 1801581 | 93507539 | 508940 | 96.82% |
| Y8 | 89870108 | 87025279 | 85330068 | 1695211 | 86568877 | 456402 | 96.83% |
