# Supplementary material for: RNA sequencing revealed the multi-stage transcriptome transformations during the development of gallbladder cancer associated with chronic inflammation
Source: PLoS One. 2023 Mar 30;18(3):e0283770. doi: 10.1371/journal.pone.0283770 (PMC10062614; doi:10.1371/journal.pone.0283770)
Supplement: S8 Table — (DOCX) [file pone.0283770.s013.docx]

**S8 Table. Primer sequences in the quantitative real-time PCR experiment**

| **No.** | **Gene** | **Primer sequences (5' to 3')** |
| --- | --- | --- |
| 1 | ENST00000555772-2F | CATTCAGACGTGGAGCAAGTTG |
|  | ENST00000555772-2R | TTTACAGACAGCGTCCATCTCACT |
| 2 | NONHSAT247740.1-F | AGAGGATTGCCAAAGAGAATGG |
|  | NONHSAT247740.1-R | GCCAACTAATCCCTGCTCAACT |
| 3 | NONHSAT104346.2-F | GGCCTCTGAAACTTGGCTTTC |
|  | NONHSAT104346.2-R | CGTCCACTCATTTCATAGGGATT |
| 4 | ENST00000648838-F | GGTTTTGCTGAGATACATGGAATTCT |
|  | ENST00000648838-R | GAGTTTTATATTTTGAGGTCCACAGACA |
| 5 | NONHSAT225391.1-F | GCAAGCTGGTTAAGTGAGCAAA |
|  | NONHSAT225391.1-R | AGGGTATGGCCTTATTCCATGA |
| 6 | NONHSAT159810.1-F | GATGGAAAGTCCAGTCCAAGGT |
|  | NONHSAT159810.1-R | CTTGAAGGATGTGCAAAATGTGA |
| 7 | IGF1-F | TCCCTTAATCTGATTTTGTTTGGAT |
|  | IGF1-R | AGAGTCCCTCCTGCAGTGTGTT |
| 8 | CYP1A1-F | CAGATCAACCATGACCAGAAGCT |
|  | CYP1A1-R | GTGAGAAACCGTTCAGGTAGGAA |
| 9 | PRKCB-F | GGAGAAACTTGAACGCAAAGAGA |
|  | PRKCB-R | CATTTCGCCCACAAGCTTTT |
| 10 | C4BPB-F | AGCTCCCAAACCAGAGTGTGA |
|  | C4BPB-R | TGGCTTCGCAGAGGTTCTTAC |
| 11 | HLA- DRB5-F | GAAAGGGCACTCTGGACTTCAC |
|  | HLA- DRB5-R | TTGAATGTGGTCATCTGCACTTC |
| 12 | SLC7A5-F | GCTCCTCCAGGGCATCTTC |
|  | SLC7A5-R | CCACCTGCATGAGCTTCTGA |
| 13 | Actin-F | CTGGAACGGTGAAGGTGACA |
|  | Actin-R | CGGCCACATTGTGAACTTTG |
